# Health coverage and what Kenya can learn from the COVID-19 pandemic

**DOI:** 10.7189/jogh.10.020362

**Published:** 2020-12

**Authors:** Polet Njeri Ouma, Abednego Nzyuko Masai, Israel Nyaburi Nyadera

**Affiliations:** 1 Faculty of Medicine, Hacettepe University, Ankara, Turkey; 2 Department of Public Health, Hacettepe University, Ankara, Turkey; 3 Department of Government and Public Administration, University of Macau, Macau; 4 Department of Political Science and Public Administration, Ankara Yildirim Beyazit University, Ankara, Turkey

COVID-19 has been a big burden to both developed and developing countries with the number of infections rising from less than one million cases in February 2020 to more than 28 million infections and 900 000 deaths by September 2020. The trend in the spread of the disease has unexpectedly overburdened even countries with stable health structures and resources. The disease has disrupted economic, social and cultural activities forcing governments to act by supporting vulnerable citizens and the sick with among other measures direct cash transfers, tax reliefs, investing more money into the economies as well as revising health insurance schemes and hospital ownership to deal with the disaster. For example, Spain nationalized all private hospitals [1] (making them accessible by public insurance) and health workers. In addition, Turkey has also included the COVID-19 cover to its universal insurance (SGK) and started providing free masks during the first weeks of the outbreak [2]. Amid the rush to find the ideal response to the pandemic, other globally trending strategies such as imposing curfews, lockdowns, and encouraging social distancing are being adopted by many countries including Kenya [1,2]. However, concerns are rising over the lack of government commitment and policy interventions on how the treatment cost of COVID -19 will be managed and the nature of support vulnerable citizens, especially low-income earners, can receive to cover costs of testing and treatment.

Perhaps such reluctance could be attributed to the low number of cases reported in most countries in Africa including Kenya. There have been numerous predictions that Africa would become the next epicenter of the disease [3-7]. However, seven months later, these worrying predictions have not come to pass. The continent has not yet felt the burden of the disease apart from South Africa and Egypt which combined constitute over 55% of the total cases in the continent. Despite the low numbers, it is too early to declare victory over the pandemic as a lack of vaccine and cure means that any country is still vulnerable to mass infections thus calling for serious reflections on the current and future state of the health sector of these countries. Kenya recorded its first case on 13th March 2020. The number of positive cases has averaged between 250 and 300 infections per day since then. This low number of daily cases can be attributed to the early response measures taken by the government to close down airports as well as partial lockdown of the capital Nairobi and the counties of Mombasa and Kilifi which are popular international tourist destinations. Cumulatively, the country has now registered over 19 000 cases as of June 30, 2020, with concerns over local transmission and the disease slowly but steadily beginning to be recorded in crowded informal urban settlements and refugee camps [8].

The fear of an uncontrolled outbreak makes the need for the government to look at the health sector holistically even more timely. More so given the constraints individuals and the health sector in the country continues to experience. Furthermore, the debate over the potentially high number of positive, asymptomatic cases not being recorded due to a low number of tests means that the government ought to prepare not only to increase and improve health care infrastructure but also effective means through which treatment will be accessible for people from all economic and social backgrounds. This should start with reforms in the national health insurance (NHIF), increasing the number of ICU beds given that currently there are less than 600 ICU beds in the whole country and the recruitment and distribution of more health workers across the country. This means that the government should work towards flattening the curve as well as restructuring the health system to a more reliable and effective sector.

## A TROUBLED PAST IN KENYA’S HEALTH SECTOR AND THE COVID -19 THREAT

Since Kenya attained independence in the early 1960s, health care has remained a privilege of the few who can afford private hospitals or treatment abroad. For the majority who are left to depend on the public hospitals, their experience has not always been smooth [9]. As a country, Kenya has also had to struggle with persistent diseases such as Malaria, TB, HIV/AIDS, and measles, other problems such as corruption in the health sector, inadequate allocation of resources, and ineffective policies making it almost impossible to enjoy quality health care. Frequent strikes by workers in the health sector, expensive prescriptions, the unattained ratio of health practitioners to population, and unequal distribution of health facilities across the country have been the norm. These challenges have weakened efforts to achieve the 3rd objective of the Sustainable Development Goals (SDG) in Kenya, which seeks to ensure individuals benefit from healthy living and well-being despite the government’s vision of attaining this goal by 2022. Of importance, is the lack of access or unaffordable health insurance that has left many people at the mercy of relatives and friends whenever hospitalized. While dependency on close members of the family and friends has been a popular trend in dealing with the gaps in the health sector, such collective action can quickly be challenged by situations such as the COVID-19 pandemic.

The rapid spread of the disease and its negative impact on the economy could undermine efforts to support others in offsetting their health bills. As of June 2020, the cost of treating a COVID-19 patient is up several thousand dollars meaning that a health system that has been underfunded, understaffed, and riddled with corruption would disintegrate if the pandemic were to lead to mass infections. This disturbing revelation means that policymakers in Kenya should not only look at curbing the spread of Coronavirus but also strive to restructure the health sector in a manner that it would be able to withstand any serious threats from the current and future health crises. There have certainly been some previous efforts to reform the health sector [10]. However, such efforts left many gaps that need to be addressed sooner than later. Previous reforms in the health sector and more specifically the provision of health insurance overlooked the need to address the unique challenges that could deny the unemployed and those who work in the informal sector from accessing health coverage. These reforms also overlooked the challenges within the health facilities in respect to unavailable, insufficient, outdated, and non-functioning equipment as well as staffing and the welfare of health workers.

## KENYA’S EFFORTS TOWARDS THE PROVISION OF UNIVERSAL HEALTH COVERAGE

The government of Kenya made changes to its national insurance scheme and introduced the National Hospital Insurance Fund (NHIF) cover in 2004 as a means of facilitating access to cheaper health in the country in line with the World Health Organization’s Universal health coverage goals [11]. However, since it was introduced, the NHIF has struggled to cater to the growing population and the growing health care needs of Kenyan citizens. According to the Ministry of Health (MoH Kenya) (2019), only 11% of Kenyans are covered by this insurance program leaving the majority of the population (89%) without the government-subsidized health plan. With over 70% of the Kenyan workforce working in the informal sector, the majority of them are either not eligible or cannot be able to afford the premiums set by the government to maintain health insurance provision. Chuma & Maina observe that since the majority of Kenyans access health care through- out-of-pocket payment, it means that many of them will avoid going to the hospital unless they are in advance stages of the disease [12]. Given the characteristics of COVID -19 such wait could result in a catastrophic spread of the disease.

**Figure:**
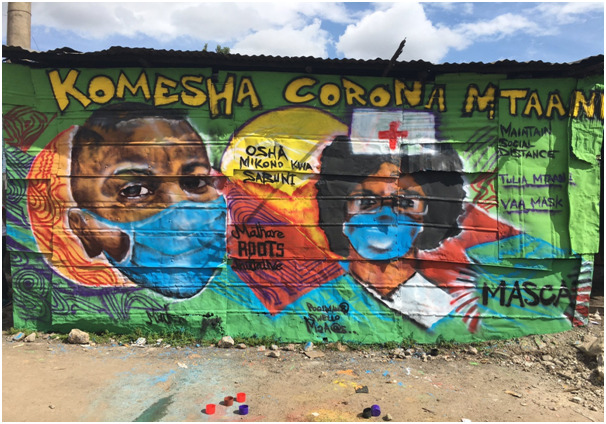
Photo: Graffiti in Mathare, one of Nairobi’s informal settlements sensitizing the community on COVID-19 (from the authors’ own collection, used with permission).

Financing management of communicable and non-communicable diseases such as Malaria, TB, and HIV/AIDS in Kenya is largely dependent on donor funds from international organizations, non-governmental entities, and contributions from other countries [13]. While contributions from these non-state actors have led to a decline in the prevalence of infectious diseases, it has, in turn, led to reluctance on the part of the government in allocating resources to the health sector. This has led to years of under-investment of the health sector, weakening health systems and structures, and raising concern as to whether the public health infrastructure can withstand a potential overburden in the wake of the COVID-19 pandemic with the majority of Kenyans facing uncertainty due to lack of health coverage.

As observed in countries such as the United States, minorities, and marginalized communities that did not have adequate health insurance were disproportionately affected by the pandemic. In Kenya where the majority of the population lacks access to health insurance, a large proportion of the citizens, therefore, remain vulnerable to the impact of a possible high prevalence of COVID-19.

The cost of COVID-19 testing in Kenya has not yet been included in the NHIF with only a few people living in hotspots areas receiving free COVID-19 testing with the rest having to pay an estimated cost of US$100 (Ksh 10 825.00) [14]. This is a difficult undertaking in a society where more than 40% of the population survives on less than US$2 (Ksh 216.31) a day. Lessons from the COVID -19 pandemic should encourage the government to pull resources even if it means cutting entertainment budget and other less essential expenditures in government to expand the NHIF scheme to cover more people, more services, and treatments(which would include but not limited to COVID-19 testing and management). This would consequently result in the aversion of a looming crisis in the health care system for not only COVID-19 pandemic but also future epidemics that might affect the country.

Kenya’s expenditure on mega projects such as infrastructure development, dams, military, and allowances for high ranking government officials need to be revised to ensure health is given more attention. For the last decade, the government’s budgetary allocation to health has averagely stood at 9% despite signing the Abuja declaration, which requires signatories to allocate 15% budgetary allocation for health [15]. This 9% of the budget that is allocated to health is further shared among the 47 counties. These funds end up being absorbed in recurrent expenditure with little left for the development of health facilities and the acquisition of medicine and equipment. With inadequate funding, the vision of the current government to achieve universal health care by 2022 seems far-fetched as efforts to introduce the universal health care program have not gone beyond the pilot stage.

An additional challenge the health sector is facing is corruption which is a nuisance that often lingers in most public projects. Citizens, therefore, remain skeptical about enrolling in the NHIF for fear of losing their investments. This means that the government needs to make urgent changes in the health sector as well as reform its image and rebuild trust with the citizens. The most recent example of mistrust is the management of the pandemic and the little transparency in the use of funds donated to tackle the pandemic. This is worsened by the alleged disappearance of donations by Chinese philanthropist Jack Ma, therefore reflecting on the historical trend of misappropriation of public health funds and resources [16].

Health reforms will ensure that resources are channeled to the health sector to help ease access to health care services and ensure that health practitioners are well equipped and well protected. At the beginning of the pandemic, a document circulated among health care providers warning them not to attend to patients in case their respective hospitals did not provide them with personal protective equipment. Although the government assured Kenyan health workers that there were enough PPE, most hospitals could not provide doctors with masks and personal protective equipment. As of April 2020, some Kenyan nurses refused to attend to coronavirus patients in protest over gear shortages. Nurses in western Kenya’s Kakamega county ran away when patients with Corona virus-like symptoms came to their hospitals while nurses in Mbagathi hospital had a go-slow protest at lack of protective equipment. Doctors in the country have also gone on strike several times in the past 10 years demanding a better working environment, employment of more doctors to ease doctor-patient ratio, higher salaries, and improvement of dilapidated public health facilities. These challenges are only examples of years of mismanagement in the health sector that has also spun into the mismanagement of the NHIF.

The crisis in the health sector needs to be taken seriously given that experiences in other countries have shown that pandemics may result in massive disruption and loss of lives. The need to address the shortage of health equipment in the country cannot be emphasized enough as equipment for proper diagnosis of ailments like cancer is unavailable. Kenya as a country has only 1 PET CT scan and a waiting period as long as six months to a year with more than 3000 oncology cases for each oncologist working in the public hospitals [17]. Moreover, most of the available equipment is in bad condition since the hospitals lack enough resources and expertise to repair broken machines or service those that are working. Mbagathi, a level 5 hospital has only one CT scan that overheats whenever used hence can only be used on two patients daily, and only three public hospitals in Kenya have an MRI machine. While NHIF has of late included a list of new benefits covering some chronic diseases like hypertension, diabetes and US$ 250 (Ksh 27 038.72) per session of chemotherapy and up to US$150 (Ksh 16 223.23) for MRI scans, the gap remains wide for many low-income earners given that an MRI scan costs roughly around US$300 (Ksh 32 446.46). This pushes for more policy reviews to explore how best NHIF would benefit the average Kenyan as the country moves towards universal health care.

## POLICY RECOMMENDATIONS

The COVID-19 pandemic has had lots of negative impacts on communities globally. It serves as a wakeup call for most governments to ensure that their citizens are well covered from potential catastrophe in the future. These important lessons seem to evade current measures being put in place to deal with the pandemic. Developing countries ought to consider health as an important component of national security and therefore treat any threat to public health as an emergency and in this case, not only by responding to curb the spread of the virus but also deal with other indirect consequences of the virus. For example, the mental health and psychological stress that is on the rise can be attributed to a lack of health insurance. Concerns over the health system’s capacity to adequately deal with the health crisis on the scale of the COVID-19 pandemic as well as the lack of social support systems that has seen many people end up in lockdown without adequate resources to sustain themselves during this period. This essay, therefore, recommends the following short, medium, and long-term interventions for Kenya’s health sector reforms.

1. The government needs to supplement the treatment of COVID-19 for all patients. Such a measure will ensure that some of the most vulnerable members of societies such as slum dwellers and low-income earners are guaranteed treatment. The cost of treating COVID-19 is extremely expensive, furthermore, the economic disruption due to the lockdown measures has interfered with the incomes of many people. This is a short term measure that will reassure the citizens that they have a dependable government. The government offering to supplement treatment of patients would mean that there would be a much quicker solution to the pandemic as those who will be infected would end up in the care of professionals and not family members who may end up spreading the disease further.
2. Streamlining service delivery in the public health sector. While Kenya has had a fair share of endemics, much of the responsibility of dealing with these diseases have been left to international organizations and non -governmental organizations with limited input from the government. These organizations have their limitations and fully depending on them undermines service delivery in the health sector. The government needs to take bold steps and increase more resources towards establishing sufficient and well-equipped health facilities as well as allocating sufficient resources towards research in the sector.
3. Related to the above is the question of corruption. Corruption can, in itself, be considered a crisis in the country. Numerous reports of corruption at the health ministry and the National Hospital Insurance Fund leaves no doubt as to why the country’s health sector is struggling. However, the importance of a healthy nation in creating a wealthy nation cannot be emphasized enough. Dealing with corruption in the health sector needs to be swift and decisive. Perpetrators of corruption should not be left to go unpunished as this sets a bad example on the protection of public resources.
4. Making universal healthcare truly universal. The government of Kenya in 2018 launched a pilot Universal Health Care (UGC) targeting 0.5% of the population (approximately 3.2 million people). Two years after the pomp and color that characterized the launch of the pilot program in the city of Kisumu, the government is yet to roll out this much-needed program. Challenges that have previously undermined access to national health coverage such as lack of transparency, access to facilities that accept the national insurance and even in places where they are accepted, key medications are not always available forcing many insurance holders to turn to private pharmacies for the medication costing them a lot more money. Universal health care means that the government should make it easy for anyone to access reasonably good quality healthcare without too many obstacles.
5. More importantly, emphasis needs to be placed on increasing recruitment and welfare of health personnel to enable efficiency in the health sector. Dealing with the periodic strikes and brain drain that has become a new normal in the health sector by ensuring health workers are well paid, offered proper protective gear that they work in a safe environment. This should be accompanied by increased distribution of health practitioners across the country to open up access. In addition, the government needs to seek the services of other related practitioners such as social workers and community development officers to supplement the work of medical workers in remote areas.

This brief note sought to examine the relationship between COVID-19 and the health sector in Kenya. It examined how the national health insurance program has been a let-down for millions of Kenyans who either are not eligible to apply, cannot afford the premiums or are simple victims of a poorly managed health system. The authors argue that the current pandemic is an eye-opener for what can be a historic reform process in Kenya’s public health system. The paper calls for not only reforms in medical spheres, but also legal, administrative, and academic sectors to ensure that health is elevated to acceptable levels. Effective global health can be achieved if national public health is well maintained. To achieve good national public health, individual health must be well protected, and to do so, individual health insurance coverage must be made accessible and effective.
